# Nickel and Ferrocene as Catalyst Candidates to Promote an Effective Oxygen Evolution Reaction

**DOI:** 10.1021/acsomega.5c00165

**Published:** 2025-05-07

**Authors:** Jose M. Abad, María Victoria Martínez-Huerta, Jesús Cebollada, Raquel Sainz, Marcos Pita, Antonio L. De Lacey

**Affiliations:** 83076 Instituto de Catálisis y Petroleoquímica, CSIC. C/Marie Curie 2, Madrid 28049, Spain

## Abstract

The oxygen evolution reaction (OER) is the main bottleneck in water splitting and other key technologies due to its slow kinetics. The development of low-cost, highly active, stable, and more efficient electrocatalysts as an alternative to the commonly expensive and scarce Ir- and Ru-based catalysts used is necessary. The present work reports the preparation of OER catalysts by a straightforward and easy method based on the synergistic effect of Ni/Ferrocene combination that leads to high current densities, lower overpotential, high stability, and low Tafel slope in alkaline conditions. The optimized Ni/Ferrocene catalyst demonstrates exceptional performance, providing a constant potential of ∼1.51 and ∼1.65 V and overpotentials of ∼0.278 and ∼0.420 V for more than 26 and 46 h at current densities of 10 and 100 mA·cm^–2^, respectively. It represents an important and significant advance in obtaining electrocatalyst materials for OER anodes without the need for previous and tedious synthetic procedures. Only commercial chemicals with low amounts of metals are employed, thus facilitating that this Ni/ferrocene configuration could be scaled up to real devices.

## Graphical abstract

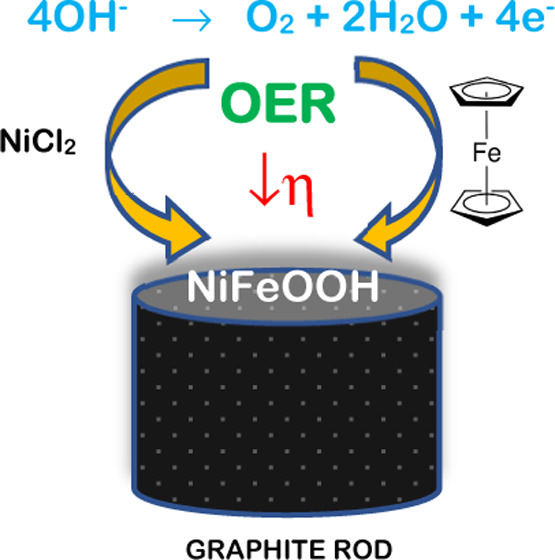

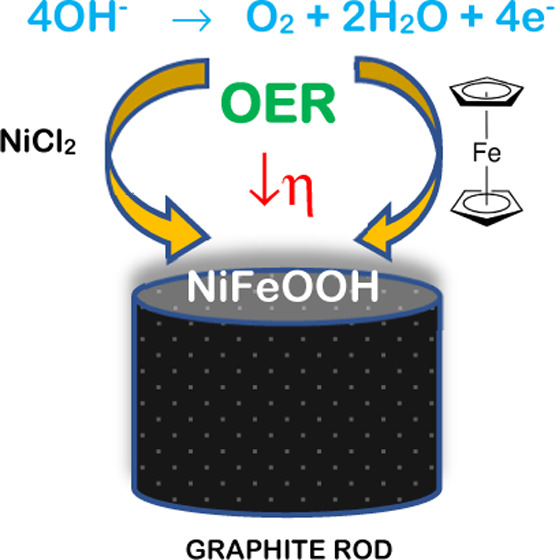

## 1 Introduction

Green hydrogen plays a key role in combating climate change and limiting global warming as a sustainable and versatile clean energy form without carbon dioxide emission into the atmosphere. Its electrochemical generation by water electrolysis allows employing electric current obtained from renewable energy sources for this purpose.^1–6^ The overall process in an electrolyzer proceeds through the oxidation of water or hydroxylic ions, depending on the pH, into oxygen (oxygen evolution reaction [OER]) at the anode and the extracted electrons are employed for the reduction of protons at the cathode to form hydrogen gas (hydrogen evolution reaction [HER]):^7^

[OER]

$$2H_{2}O\to O_{2}+4H^{+}+4e^{-}\left(\mathrm{acid}\right)$$

$$4{\mathrm{OH}}^{-}\to O_{2}+2H_{2}O+4e^{-}\left(\mathrm{alkaline}\right)$$

[HER]

$$4H^{+}+4e^{-}\to 2H_{2}\left(\mathrm{acid}\right)$$

$$4H_{2}O+4e^{-}\to 2H_{2}+4{\mathrm{OH}}^{-}\left(\mathrm{alkaline}\right)$$

The bottleneck of this process is the OER due to its slow kinetics. Therefore, a catalyst is required to overcome the high activation barrier for removing 4e^–^ per O_2_ molecule, allowing the OER at a small overpotential versus the thermodynamic one of +1.23 V (at pH 0). Research in this field has focused on the development of low-cost, highly active and stable electrocatalysts, aiming for a more efficient electrolytic OER, yielding lower overpotentials and thus reducing reaction energy consumption, which conveys an energy conversion efficiency.^8^

To date, catalysts involving noble metals of the platinum group, such as iridium (Ir) and ruthenium (Ru), their alloys and oxides, as well as their composites, occupy the top position as favorites due to their better performance in terms of low overpotential, Tafel slope, and stability.^9–14^ However, they are scarce and expensive, which hinders their practical applications, and thus, the development of noble metal-free catalysts is needed. Our aim is the development and preparation of efficient, simple, and cost-effective OER catalysts, avoiding the use of scarce precious metals and potentially applicable on an industrial scale without tedious and sophisticated synthetic procedures. As an alternative, electrocatalysts formed by transition metals (Fe, Co, Ni, and Mn) and their oxides/hydroxides/oxyhydroxides have been extensively explored due to their multivalent oxidation states (as it is been proved that the M^+2/+3/+4^ states are the active sites for OER).^15^ They show high-performance electrocatalysis with good corrosion resistance and are cheaper to obtain.^16–23^ The OER activities of these materials are highly dependent on the morphology, composition, oxidation state, the 3d electron number of their transition metal ions, the surface oxygen binding energy, and the enthalpy for the lower to higher oxide transition.^24^ Numerous advances have been made in the development of new catalysts by testing the use of different combinations of these transition metals, but most of them involve tedious synthetic procedures. Recently, we have developed a simple and straightforward method for preparing an electrocatalyst for OER in alkaline media that combines Co and Ferrocene (Fc) deposited on a carbon electrode.^25^ This configuration demonstrated a synergistic effect, achieving high catalytic activity with minimal loading of Fc and cobalt­(II) chloride. The resulting electrocatalysts proved to be highly effective for the OER, displaying excellent activity and stability, along with a low overpotential in an alkaline medium. Following this work, we report the preparation and performance of the OER of electrocatalysts formed by nickel oxide and ferrocene. Ni-based catalysts in aqueous alkaline media are low-cost electrode materials proposed as alternative electrocatalysts because of their abundance in the Earth's crust and efficient high current density operation.^26^ They display superior performance toward electrocatalytic OER activity in the order Ni > Co > Fe > Mn.^27^ In addition, nickel-based oxides and (oxy)­hydroxides are very resistant to corrosion, having higher stability than other precious metal catalysts.^28^ The higher oxidation state of NiOx (Ni^3+^) is very active for the OER, whereas Ni^2+^ is more stable. The introduction of other metals, such as Fe, enhances the OER activity by stabilizing Ni^2+^/Ni^3+^ active sites and by improving the electrical conductivity via intermediate bonds and electronic structure modification.^29–32^ Well-designed FeNi-based hybrid catalytic materials in an alkaline environment have exhibited superior OER performance comparable to benchmark OER Iridium-based catalysts.^30–39^ However, it has been pointed out that the key problem limiting the development of FeNi-based hybrid catalysts is that anodic currents cause the reconstruction of their surface upon oxidation, leading to the loss of active sites and stability attenuation.^40^ Furthermore, nickel–iron catalysts are very prone to degradation at high temperatures, making it challenging to create active nickel–iron catalysts with high surface area through traditional heat-treatment methods. To overcome this, herein, we have prepared hybrid catalysts by a simple procedure developed for the use of ferrocene combined with nickel. Ferrocene is an inexpensive and robust compound based on earth-abundant iron that has been explored as an alternative catalyst versus other metal oxides for the oxygen evolution reaction (OER) due to its unique properties. The iron center in ferrocene can undergo redox reactions, which are crucial for the process of the OER process. Additionally, ferrocene’s stability and ability to form various derivatives make it a versatile candidate for catalyst design. It has been recently reported that the surface modification of Ni­(OH)_2_ with a trace amount of ferrocene formic acid leads to OER performance enhancements.^40^ Also, in another recently published work, ferrocene has been combined with nickelocene for electrodeposition of nickel–iron-based nanostructured film using nonaqueous electrolyte and these organometallic complexes as metal sources.^41^ Although these films exhibited an efficient OER, they required a previous electrochemical deposition process for obtaining them. Therefore, our goal has been to produce porous Fc/Ni-based catalysts in a simpler way to obtain higher OER performances, such as high intrinsic activity, large surface area, and sufficient stability in oxidative electrochemical environments. Ferrocene and nickel chloride mixed with carbon Vulcan and Nafion were deposited straightforwardly on a graphite electrode (Scheme 1). The resulting catalysts were characterized by cyclic and linear voltammetry and SEM, and their surface compositions were determined ex- and in situ by Raman, X-ray photoelectron spectroscopy (XPS), and X-ray diffraction analysis (XRD).

**Scheme 1:**
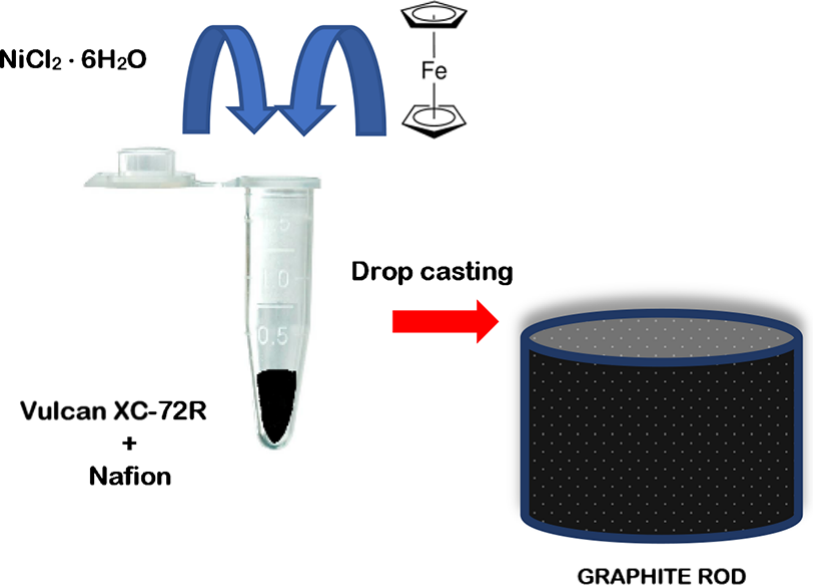
Preparation Process for a Ni/Fc-Modified Electrode Using a Facile Straightforward Approach

## 2 Experimental Section

### 2.1 Materials and Reagents

Graphite rods of 3 mm diameter (0.071 cm^2^ geometric area), low density, 99.995% trace metals basis, Nafion perfluorinated resin solution (5 wt %), ferrocene (98%), and nickel chloride hexahydrate (ACS reagent) were supplied by Sigma-Aldrich. Carbon black Vulcan XC-72R was purchased from the FuelCell company.

### 2.2 Catalytic Ink Preparation and Deposition on Electrode

The electrocatalyst preparation followed our recently published methodology.^25^ Briefly, catalytic ink was made by mixing 1.5 mg of carbon black Vulcan in a solution of ethanol: water (1:0.25 mL) and adding 5 μL of Nafion. This mixture was then sonicated for 30 min in an ultrasound bath to achieve a homogeneous ink. The final catalytic ink was subsequently obtained by adding 20 μL of the sonicated ink, 20 μL of a 10 g·L^–1^ ferrocene ethanolic solution, and 4 μL of a 100 g·L^–1^ nickel chloride hexahydrate aqueous solution, previously prepared. Following this, 2 μL of this ink was drop-cast onto the surface of a graphite electrode previously polished softly on a fine grit polishing pad (BASi), rinsed, and sonicated for 15 min in Milli-Q water. It was left to dry for 5 min at room temperature, and this process was repeated twice. The final optimized amounts loaded were 36 μg, 0.5 mg·cm^–2^ NiCl_2_·6H_2_O and 18 μg, 0.25 mg·cm^–2^ ferrocene. Other nickel and ferrocene loadings were prepared following the same procedure but employing NiCl_2_· 6H_2_O and ferrocene stock solutions of higher or lower concentrations.

### 2.3 Electrochemical Measurements

All electrochemical measurements were conducted using an Autolab potentiostat (PGSTAT 30, Eco Chemie) with a three-electrode cell using a graphite rod and a mercury oxide (Hg/HgO, handled and disposed of according to local EPA guidelines) electrode as counter and reference electrodes, respectively, in a 1 M KOH solution (pH 13.89), at room temperature. For analysis and comparison, all potential values measured versus Hg/HgO were converted to potentials versus the reversible hydrogen electrode (RHE) using the Nernst equation as follows:

$$E_{\mathrm{RHE}}=E_{\mathrm{Hg}/\mathrm{HgO}}+0.0592\mathrm{pH}+E{{}^{\circ}}_{\mathrm{Hg}/\mathrm{HgO}}=E_{\mathrm{Hg}/\mathrm{HgO}}+0.928V$$

where *E*°_Hg/HgO_ is the standard thermodynamic potential (0.1053 V) of Hg/HgO, *E*_Hg/HgO_ is the potential vs Hg/HgO obtained during electrochemical measurements, and *E*_RHE_ represents the potential vs RHE.

The reported overpotential η refers to a current density reaching 10 mA cm^–2^:

$$\eta =E(10\;\mathrm{mA}\;{\mathrm{cm}}^{-2})-E{}^{\circ}(1.23\;V)$$

The chronopotentiometric stability tests were conducted for 26 and 46 h at applied current densities of 10 and 100 mA·cm^–2^, respectively in a 1 M KOH solution. Electrochemical impedance spectroscopy (EIS) was performed on the prepared electrodes within the frequency range of 0.1 MHz to 1 Hz, using an AC potential amplitude of 10 mV and a bias potential of +1.65 V vs RHE. The Nyquist plots obtained were analyzed to evaluate the resistance of the electrocatalytic materials.

Tafel analysis of the OER was conducted through chronoamperometry measurements, focusing on the initial low current density range. The interval between step potentials was 0.01–0.02 V, and the potential was corrected for *iR* drop before plotting it against the measured logarithmic current density.

Turnover Frequency (TOF) was calculated using equation:^41^

$$\mathrm{TOF}=(j\times N_{A})/(4\times F\times n)$$ 1

wherein *j*, *N*_*A*_, *F*, and *n* are respectively the current density in A·cm^–2^ for OER measured at the overpotential of 300 mV, the Avogadro number (6.023 × 10^23^), the Faraday constant (96485 C·mol^–1^), and the electrochemically active sites for OER calculated from the integration of the cathodic redox peak of Ni^2+^/Ni^3+^ in Figure 1.

**Figure 1:**
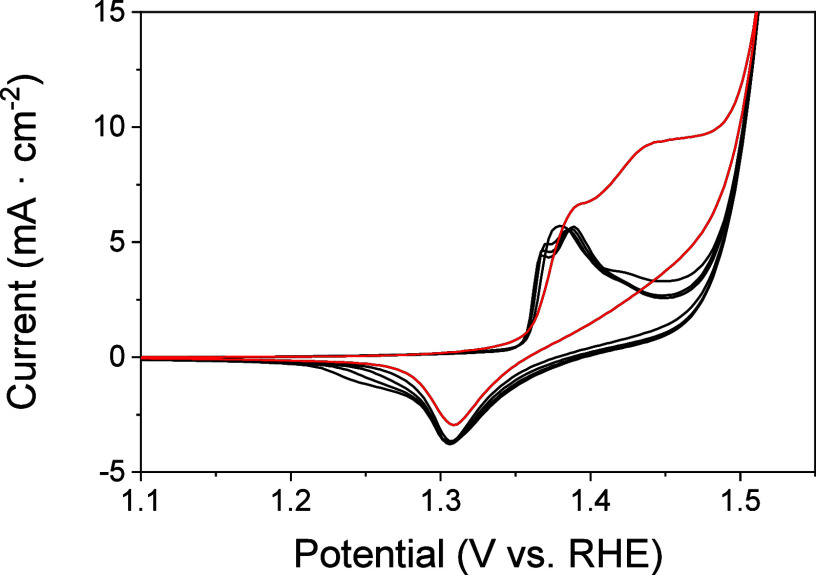
Cyclic voltammograms (CVs) of a Ni/Fc-modified electrode in 1 M KOH at a 5 mV·s^–1^ scan rate. Red and black lines correspond to the first and successive scans, respectively.

### 2.4 Catalyst Characterization

A FEI Quanta 200 scanning electron microscope (SEM) was employed to characterize the morphologies of the catalysts, and their elemental composition reported was obtained by energy-dispersive X-ray microanalysis (EDX) coupled to SEM.

Raman spectra were recorded with a Renishaw inVia Qontor instrument equipped with a cooled CCD detector, a confocal microscope, and a 514 nm Ar ion laser. Measurements were performed using a Raman Electrochemical Flow Cell (Redox.me) in 0.1 M KOH. A graphite rod was used as the working electrode, a platinum wire as the counter electrode, and Ag/AgCl as a reference electrode. An Autolab PGSTAT204 potentiostat/galvanostat was used for electrochemical measurements. Each spectrum was recorded for 50 s after the beginning of each potential step from the OCP until 1.65 V versus RHE.

X-ray photoelectron spectroscopy (XPS) data were obtained with a SPECS GmbH system equipped with a hemispherical energy analyzer, PHOIBOS 150 9MCD. A nonmonochromatic Mg X-ray source was used with a power of 200 W and a voltage of 12 kV. Pass energies of 50 and 20 eV were used to acquire survey and high-resolution spectra, respectively. These pass energies correspond to an Ag 3d5/2 fwhm of 1.6 and 1.0 eV. The peak positions of the Ag 3d5/2 and Ag MNN peaks were used for instrument calibration. The Ag 3d5/2 and Ag MNN peaks were found to be at 368.23 eV and 895.74 eV binding energy positions, respectively.

X-ray diffraction (XRD) was carried out by means of an X-Pert Pro PANalytical instrument with Cu Kα radiation (1.5418 Å).

## 3 Results and Discussion

### 3.1 Optimization of Ni/Fc Catalyst and Electrochemical Characterization

The electrocatalysts were prepared employing a facile procedure consisting of drop-casting on porous graphite electrodes a tiny amount of a catalytic ink containing different amounts of NiCl_2_·6H_2_O and ferrocene as the only metals mixed with activated carbon Vulcan XC-72R and Nafion, as described in the previous section. The even distribution was guaranteed by previous sonication of the ink to obtain a homogeneous dispersion of Vulcan and Nafion, in which nickel chloride and ferrocene were perfectly solubilized.

A preliminary study was carried out to estimate the optimal amount of ferrocene and nickel for obtaining the best performance in terms of the low OER overpotential and high catalytic activity. Figures S1 and S2 show linear sweep voltammograms (LSVs) of the OER of electrocatalysts in 1 M KOH, prepared with different loadings of nickel and Fc, respectively. It was found that the lower OER overpotential and higher current are obtained with 36 μg (0.5 mg·cm^–2^) of NiCl_2_ · 6H_2_O and 18 μg (0.25 mg·cm^–2^) of Fc. These amounts were chosen for successive experiments. Larger or lower amounts of nickel or Fc resulted in higher OER overpotentials and lower electrocatalytic currents. Cyclic voltammetry was employed to characterize and elucidate the different species involved during the transformations of the OER process and their transformations. Figure 1 shows the CVs for the electrodes modified with Ni^2+^/Fc measured in 1 M KOH at 5 mV·s^–1^. It is expected that Ni^2+^ from the NiCl_2_ reacts initially in an alkaline medium with hydroxyl ions forming nickel hydroxide (Ni^2+^ + 2OH^–^ ⇄ Ni­(OH)_2_), thus the anodic and cathodic peaks appearing during the first scan at 1.39 and 1.31 V, respectively, correspond to the redox couple Ni­(OH)_2_/NiOOH.^31,42^ This reaction proceeds in alkaline electrolytes as β-Ni­(OH)_2_ + OH– ⇄ β-NiOOH + H_2_O + e^–^.^43–45^ Therefore, this oxidation process leads to the in situ electrochemical formation of nickel oxyhydroxide on the surface.^45–47^ Successive scans at low scan rate show a shift in the Ni­(II)/Ni­(III) redox potential with the appearance of a new peak at 1.37 V besides a cathodic wave at 1.24 V and a decrease of the anodic peak at 1.44 V associated with the oxidation of Fc. These changes can be attributed to the transformation of β-NiOOH to the ordered and compact γ-NiOOH, besides an electrochemical reconstruction process due to the interaction of Fe–Ni heteroatomic pairs.^36,39,40^ Those pairs of quasi-reversible peaks, the one observed in the first scan and the other observed in successive scans, have been proposed to correspond to sites in the structure with different amounts of Ni/Fe ratios.^37^ They have half-wave potentials, *E*_1/2_ = (Ep_c_ + Ep_a_)/2, of 1.35 and 1.31 V (where Ep_c_ and Ep_a_ are the cathodic and anodic peak potentials, respectively), and a peak-to-peak separation (ΔEp = Ep_a_–Ep_c_) of ∼80 and 130 mV, respectively, at a scan rate of 5 mV·s^–1^.

### 3.2 Electrochemical Evaluation for OER Activity of the Ni/Fc Catalyst

Electrochemical evaluation of the performance of the OER for the Ni/Fc-modified electrodes was carried out by linear sweep voltammetry measurements (Figure 2). In the first scan, the anodic peaks corresponding to the transformation of Ni­(OH)_2_/NiOOH and Fc oxidation can be seen (Figure 2, inset), which shift to lower potential while the Fc peak disappears at the second scan, similarly to that observed above in the CVs. In addition, a high anodic current due to Ni^3+^(O)–OH + OH^–^(aq) ⇄ Ni^2+^–OH + O_2_(*g*) + e^–^, was measured, leading to a large number of bubbles of oxygen gas evolving on the electrode surface due to the water oxidation reaction. The onset potential of the OER was 1.47 V with a remarkable overpotential at 10 mA·cm^–2^ of 0.276 V relative to the theoretical value of 1.23 V vs RHE.

**Figure 2:**
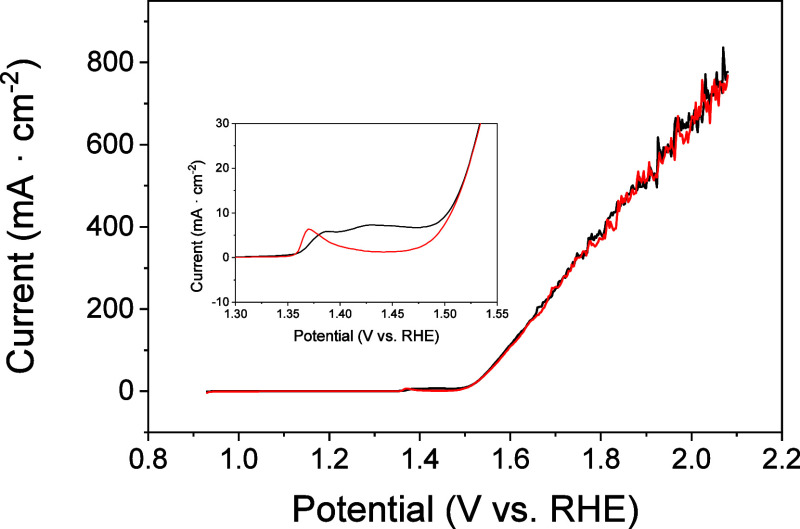
Linear sweep voltammograms of a Ni/Fc-modified electrode in 1 M KOH at 5 mV·s^–1^: first scan (black line); successive scans (red line).

Control experiments were also carried out by preparing catalysts without Fc or Ni or with only Vulcan. As shown in Figure 3, for all control configurations, higher overpotentials were required, and lower OER activities were obtained than those for Ni/Fc-modified electrodes. This result demonstrates the synergistic effect between Ni/Fc, which can be attributed to the formation of Ni/Fe oxyhydroxide [Ni­(Fe)­OOH].^35,39^ This is in agreement with what we previously reported for Co/Fc-modified electrodes, but it is remarkable that in the present case, Ni/Fc requires a lower overpotential than Co/Fc (0.310 V).^25^ Furthermore, the high current density exhibited in the LSV normalized by the mass of NiCl_2_·6H_2_O and ferrocene deposited on the electrode reflects the efficient catalysis obtained for the OER process (Figure S3A) using a minimal amount of catalyst.

**Figure 3:**
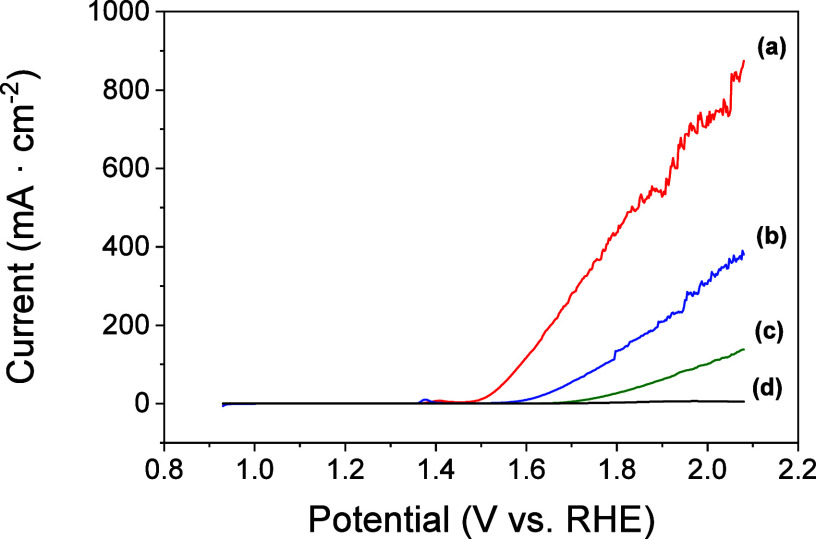
Linear sweep voltammograms in 1 M KOH of: (a) Ni/Fc-modified electrode; (b) Ni-modified electrode; (c) Fc-modified electrode; (d) only Vulcan-modified electrode. All graphite electrodes were prepared as described in the procedures (Supporting Information). Scan rate, 5 mV·s^–1^.

The ferrocene group appears to have a significant synergistic effect, although the mechanism leading to superior OER activity for Ni/Fc catalysts is still rather unclear. Reported DFT calculations^48^ have concluded that ferrocene units can act as mediators to facilitate electron transfer, thus contributing to the outstanding intrinsic activity observed. However, another plausible mechanism is the incorporation of iron from ferrocene. It has been indicated that iron in Ni/Fe catalysts acts as an alloying element and a strain on the lattice, stabilizing the higher oxidation states of nickel (Ni^3+^, Ni^4+^), inducing charge transfer to Ni sites, and leading to a superior OER activity.^30,49–53^ In this way, the ferrocenium cations generated anodically during OER could suffer decomposition into Fe^3+^ and Fe_2_O_3_ in the presence of oxygen, as reported by Singh et al.^54^ Subsequently, it can be incorporated into the structure of NiOOH to give Ni_1–*x*_Fe_*x*_OOH catalysts and acting these Fe sites as active sites for OER. Furthermore, it has also been demonstrated that the incorporation of Fe to form a mixed Ni–Fe oxyhydroxide leads to an increase in conductivity relative to NiOOH, contributing partially to the enhanced OER activity.^30,36^ More recently, Liu et al.^55^ have provided a comprehensive understanding of how Fe and Ni work together in NiFeOOH to enhance the OER performance, offering new insights into the synergistic effects among different metals. Fe-doped NiOOH (NiFeOOH) has been found to have a multisite dynamic synergistic reaction mechanism, where Fe, O, and Ni atoms act as active sites in a dynamic and sequential manner during the OER process. The study highlights the role of electron channels related to the magnetic states among Fe–O–Ni, which helps in decoupling the OER sites from the oxidation reaction sites. This decoupling is crucial for efficient catalysis.

Additionally, electrochemical impedance spectroscopy (EIS) was also performed to estimate the electron transfer resistance, *R*_et_. Figure S4a depicts the Nyquist plot for the Ni/Fc electrocatalyst, where a low *R*_et_ value of 4 Ω was obtained. Similarly, ∼11 Ω was estimated for Ni-catalysts without Fc, which is less than that obtained for Fc in the absence of Ni species (446 Ω) and much less than that previously obtained for a Vulcan carbon electrode (∼2500 Ω), indicating that R_et_ is greatly improved when Ni^2+/3+^ is present. Also, the uncompensated resistance value (*R*_u_) for the Ni/Fc catalyst was determined to be 11 Ω. This value was employed to compensate for the *iR* drop for the previously acquired LSV of the Ni/Fc-modified electrode (Figure S4). Consequently, the overpotential η at 10 mA cm^–2^ (0.23 V) was shifted to a lower value.

The electrochemically accessible surface area (ECSA) was also estimated from the Nyquist plot (Figure S4) by calculating first the double layer capacitance *C*_dl_ and subsequently ECSA using the equations:

$$C_{\mathrm{dl}}=\frac{1}{2\pi f\mathrm{Ret}}$$ 2

$$\mathrm{ECSA}=\frac{C_{\mathrm{dl}}}{C_{s}}$$ 3

where *f* is the frequency at the peak of the semicircle at which the imaginary part of impedance (−Z̈) is maximal and *C*_*s*_ is the specific capacitance, whose value of 300 μF·cm^–2^ was taken from the literature.^41^ Values of 1.92 × 10^–3^ F and 6.4 cm^2^ were determined for *C*_dl_ and ECSA, respectively. This large area is in agreement with the high catalytic performance for the OER observed for the porous Fc-Ni catalyst. The value of the ECSA was used to normalize the current obtained for OER by LSV (Figure S3B).

### 3.3 XPS, Raman, and XRD spectroscopy characterization

In support of the above hypothesis, the Ni-Fc catalysts were also characterized by XPS and Raman measurements before and after the electrochemical OER experiment. The XPS spectra for Ni-Fc catalysts before any electrochemical process was measured and after testing the performance of the OER are shown in Figure 4. High-resolution core Ni 2p spectra show spin–orbit splitting into 2p1/2 and 2p3/2 components with one main peak located at 857.8 eV for the as-prepared catalyst-modified electrode, which is associated with the ionic interaction of nickel­(II) ions with fluoride contained in the Nafion polymer in alkaline hydroxide.^56^ In addition, the deconvoluted spectra (Figure 4) also showed a band at 856.3 eV corresponding to the binding energy of Ni­(OH)_2_ formed initially. This band is the main one obtained after OER, which could be consistent with either remaining Ni­(OH)_2_ or more, presumably due to the NiOOH phase generated during the electrochemical process, as previously observed in the voltammetry measurements and further detected by Raman microscopy (vide infra).^57–59^ On the other hand, negligible broad XPS energy bands were obtained for Fe (Figure S6), as a consequence of its low content in the catalyst. Thus, it prevented their deconvolution and identification. Also, a low signal was obtained for a Ni catalyst in the absence of Fe, where the Ni 2p spectrum after the performance of the OER showed barely noticeable XPS energy bands (Figure S6B).

**Figure 4:**
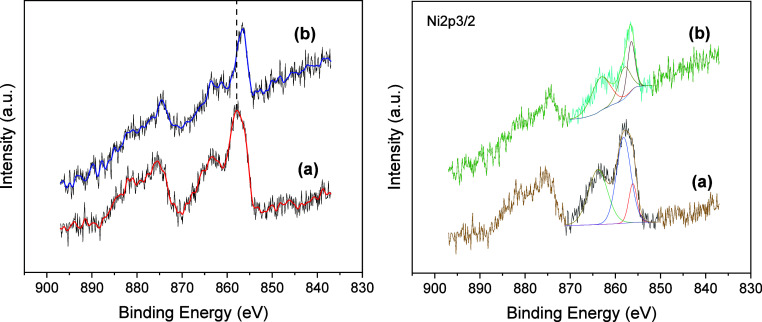
High-resolution XPS core spectra for Ni/Fc-modified electrodes at the Ni 2p energy region: electrodes as-prepared before measuring any electrochemical process (curve a) and after studying their OER performance (curve b). The right graph shows the deconvoluted XPS spectra.

We also carried out measurements by Raman spectroscopy ex situ and in situ. Figure 5 shows the spectra collected for Ni-Fc catalysts as-deposited before any electrochemical process and after testing the OER performance in 0.1 M KOH. The Raman spectrum before OER shows only the characteristic G and D bands associated with graphite located at ∼1584 and ∼1357 cm^–1^, respectively, besides the 2D band at ∼2718 cm^–1^.^60^ By contrast, two pairs of bands appeared at 474 and 554 cm^–1^ after the electrochemical OER experiment, which are associated with the bending and stretching vibrational modes of the Ni–O bonds in NiOOH, respectively. This result is in agreement with that previously reported for NiFeOOH catalysts, where these two vibrations are known to show a high Raman cross section due to resonance effects.^31^ Furthermore, the ratio of the intensity of the 474 cm^–1^ band to that of the 554 cm^–1^ is higher, which is expected for γ-NiOOH rather than for β-NiOOH.^31,61,62^ It confirms that electrochemical transformation to γ-NiOOH occurred during the OER.

**Figure 5:**
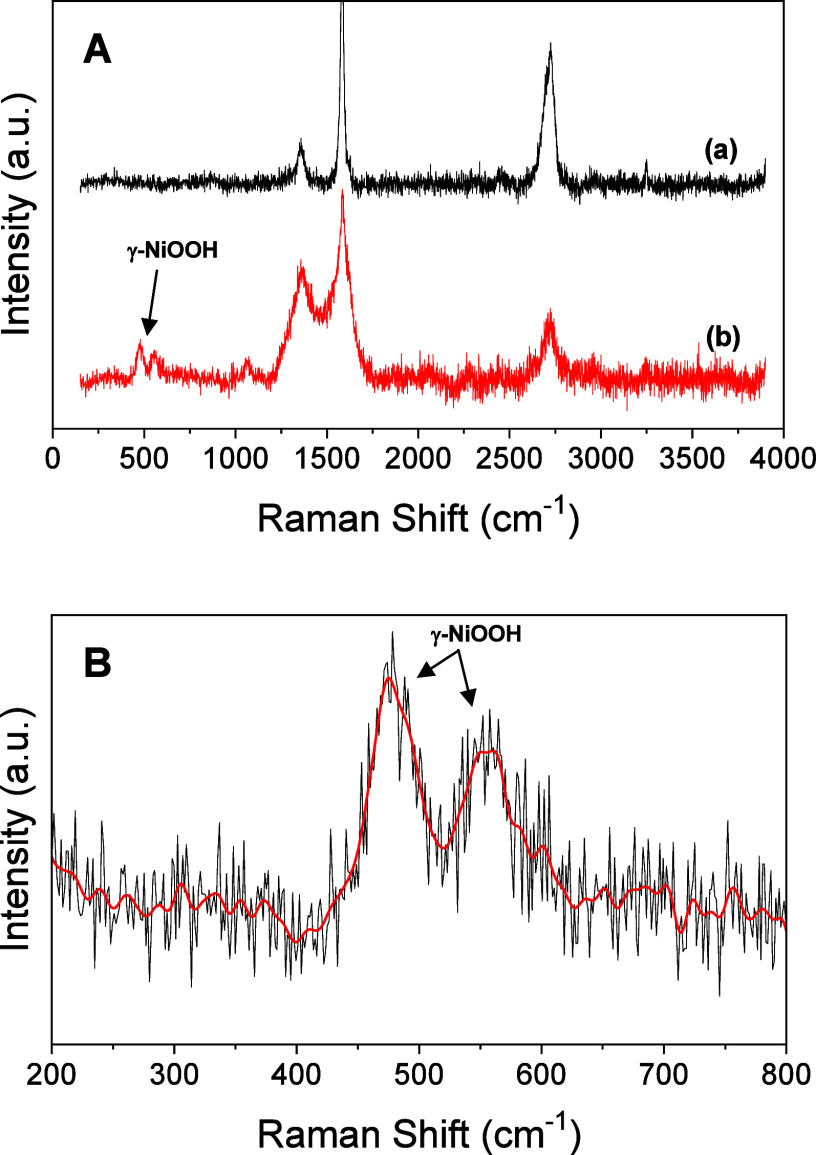
(A) Raman spectra collected for Ni/Fc-modified electrodes: (a) as-prepared before any electrochemical process; (b) after studying their OER performance. (B) zoomed region of spectrum at low wavenumbers where peaks are observed after OER.

To gain more insight into the mechanism of electrochemical transformation that occurred during the process, we carried out in situ Raman microscopy measurements for electrodes with different catalyst configurations at different applied redox potentials. Figure S7 shows a series of Raman spectra acquired in KOH 0.1 M for the Fc catalyst without nickel. No spectral features and changes were observed until a potential of 1.30 V was applied, where the apparition of a main band at 670 cm^–1^ starts for Fe–O, corresponding to the formation of γ-FeOOH.^63^ By contrast, Raman spectra of the Ni-Fc catalyst (Figure 6) showed at 1.25 and 1.30 V characteristic bands with a defined crystalline lattice, which have been reported to be associated with nickelocene formation.^64–67^ The band at 1103 cm^–1^ corresponds to the ring breathing mode of the cyclopentadienyl (Cp) rings, whereas the band obtained at 1058 cm^–1^ is typically associated with the C–C stretching vibrations within the cyclopentadienyl (Cp) rings of nickelocene, and the bands at 392 and 309 cm^–1^ are due to nickel-ring stretching vibrations. These results suggest that Fc is decomposed due to its instability in the presence of oxygen into Fe^3+^ and cyclopentadienyl rings, the latter would react with Ni^2+^ to form temporally nickelocene. By applying higher potentials, these bands disappear due to degradation of nickelocene into Ni oxide, beginning the apparition of bands at 474 and 554 cm^–1^ corresponding to the formation of γ-NiOOH.^31,61,62^

**Figure 6:**
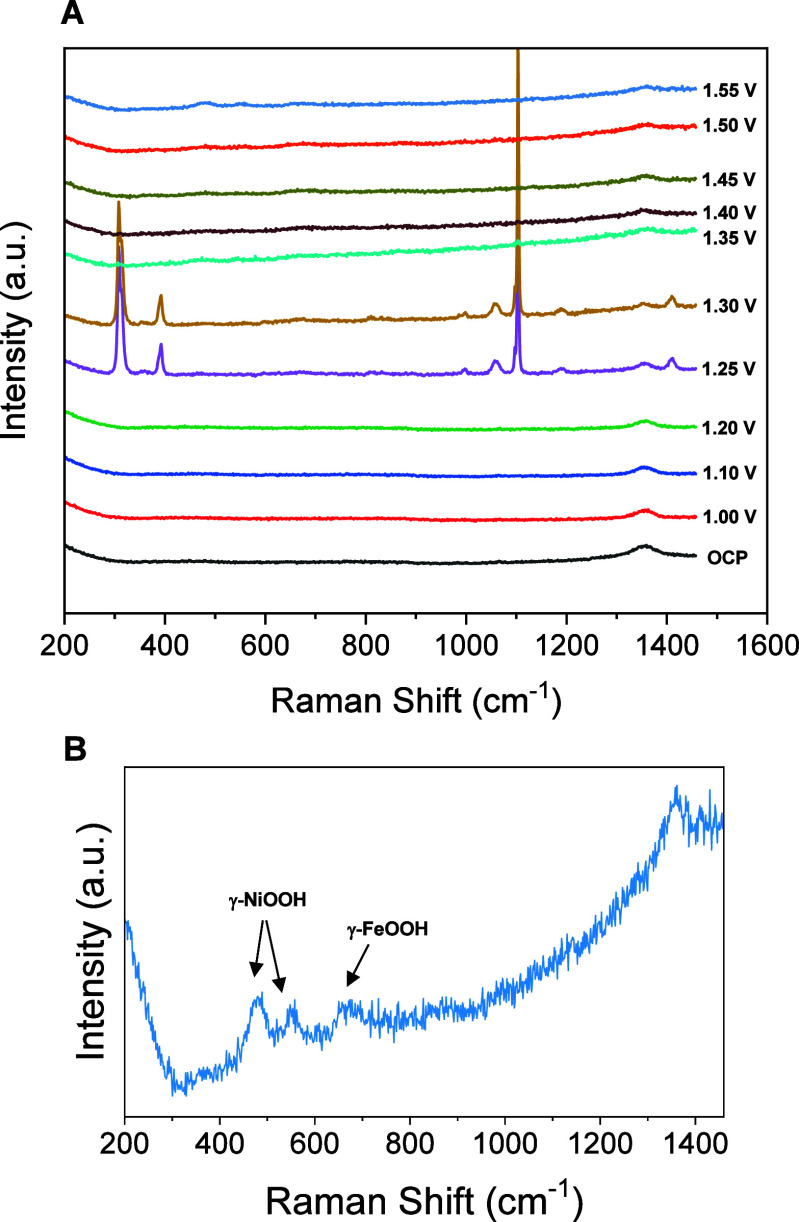
In situ Raman spectra collected for Ni/Fc-modified electrode as a function of potential applied vs RHE in 0.1 M KOH (A) and (B) zoomed spectra obtained at 1.55 V.

The band can also still be observed slightly at around 670 cm^–1^ of γ-FeOOH. Carrying out a second round of applied potentials (Figure 7), those γ-NiOOH bands are clearly observed starting at a potential of 1.45 V, being maximum at 1.50 V, while the band of γ-FeOOH has disappeared as a consequence of Fe atoms dissolution into the electrolyte with the increasing potential.^37^ This potential value and behavior match perfectly with that observed at 1.39 V by cyclic voltammetry and LSV characterization in 1 M KOH (it has to be taken into account that the 0.059 V lower potential measured in 1 M KOH is expected for a change of one pH unit). As previously shown, successive LSV and CV scans cause a decrease and final disappearance of the peak corresponding to Fc oxidation, with a simultaneous shift of the peaks corresponding to NiOOH. All of these results corroborate the transformation of Ni-Fc into NiFeOOH and its further structural evolution from β-NiOOH/γ-FeOOH to γ-NiOOH.

**Figure 7:**
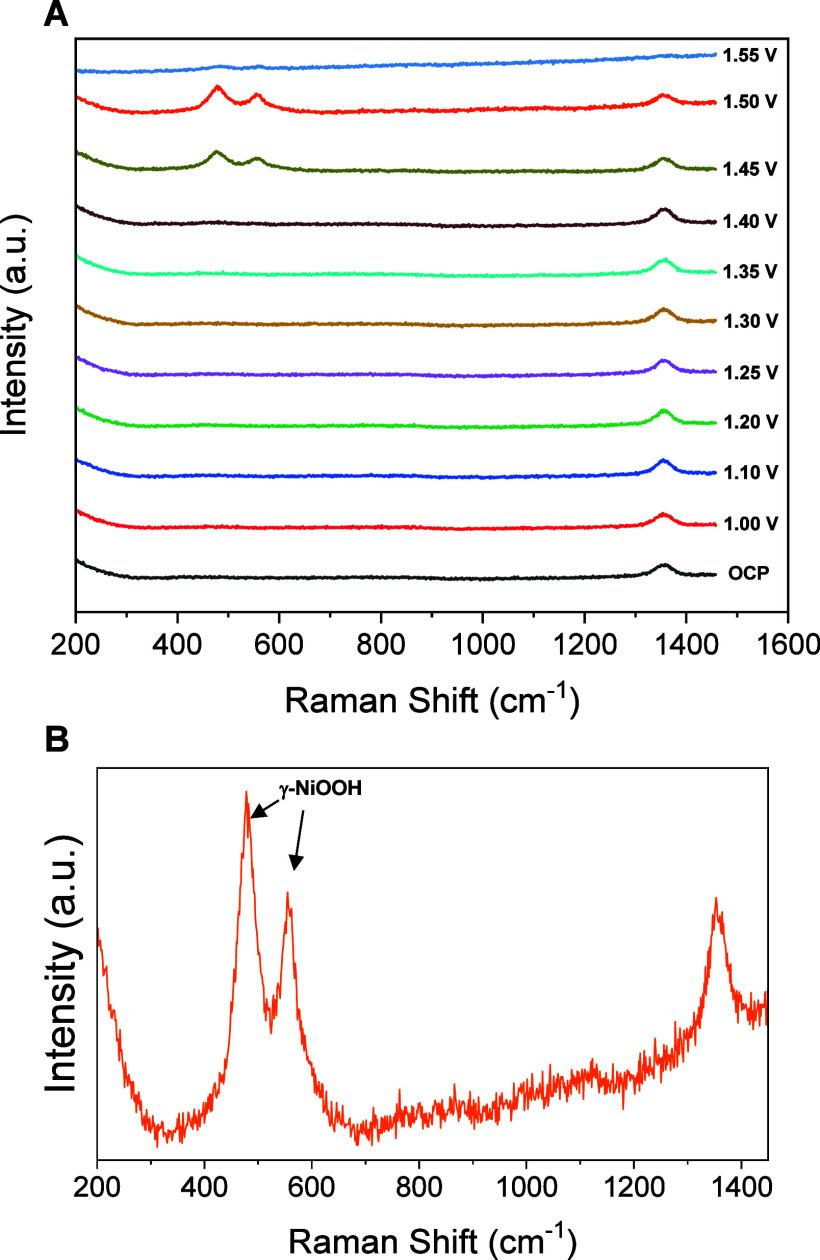
*In situ* Raman spectra collected for Ni/Fc-modified electrode as a function of applied potential vs RHE in 0.1 M KOH (second round) (A) and (B) zoomed spectra obtained at 1.50 V.

XRD characterization carried out of the Ni/Fc-modified electrode as-prepared and after studying their OER performance (Figure S8A) showed only diffraction peaks similar to those obtained for the graphite electrode containing only Vulcan (Figure S8B). These bands were characteristic of graphite structure without the presence of new crystalline phases that might have formed during the OER, indicating possible degradation or transformation of the catalyst. The absence of bands associated with FeNiOOH may be due to it exhibiting an amorphous structure since its synthesis is reported in such a form.^68^ Its amorphous nature can enhance its catalytic activity due to the increased availability of active sites and its structural flexibility.

### 3.4 Morphological Characterization of Ni/Fc catalyst

SEM measurements were carried out for characterization of the surface morphologies of the as-prepared Ni/Fc catalyst-modified electrode and after studying the OER performance (Figure 8). In both cases, the SEM images obtained at different magnifications show a spongy porous structure on the graphite electrode being more homogeneously distributed in the catalysts after the OER experiments. Energy-dispersive X-ray spectroscopy (EDX) elemental analysis was employed to identify and semiquantitatively quantify the presence of Ni and Fe elements in different areas on the surface. Ni and Fe were found for catalysts after OER with an average ratio of Ni/Fe ≈ 1.8–2.5 (Figure S9). Their spatial distribution was determined by EDX elemental distribution mapping, showing that the nickel and iron elements were uniformly dispersed over the electrode surface (Figure 9).

**Figure 8:**
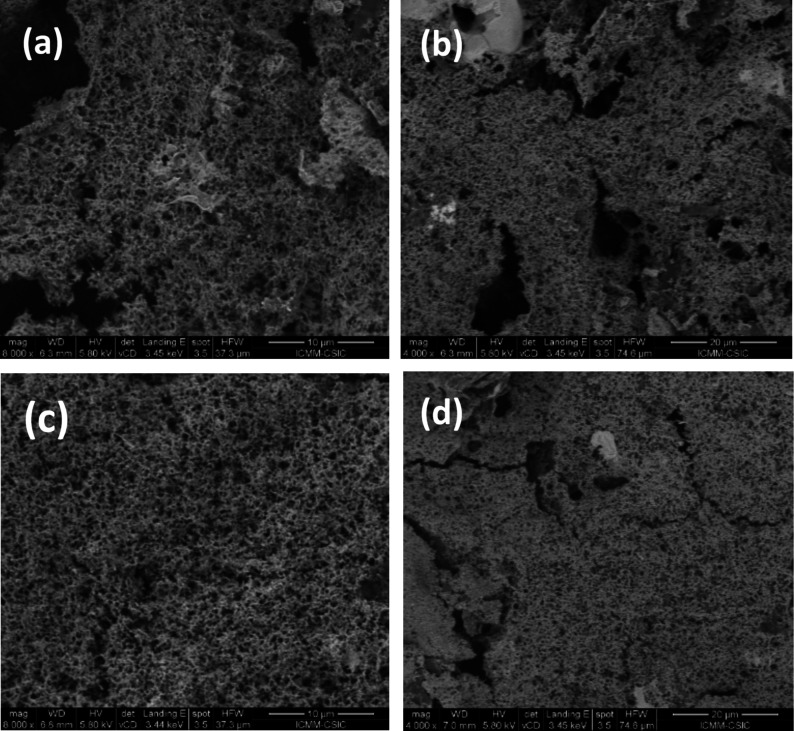
SEM images of Ni/Fc catalyst electrode surfaces at different magnifications: (a, b) as-prepared; (c, d) after studying OER performance in 1 M KOH solution.

**Figure 9:**
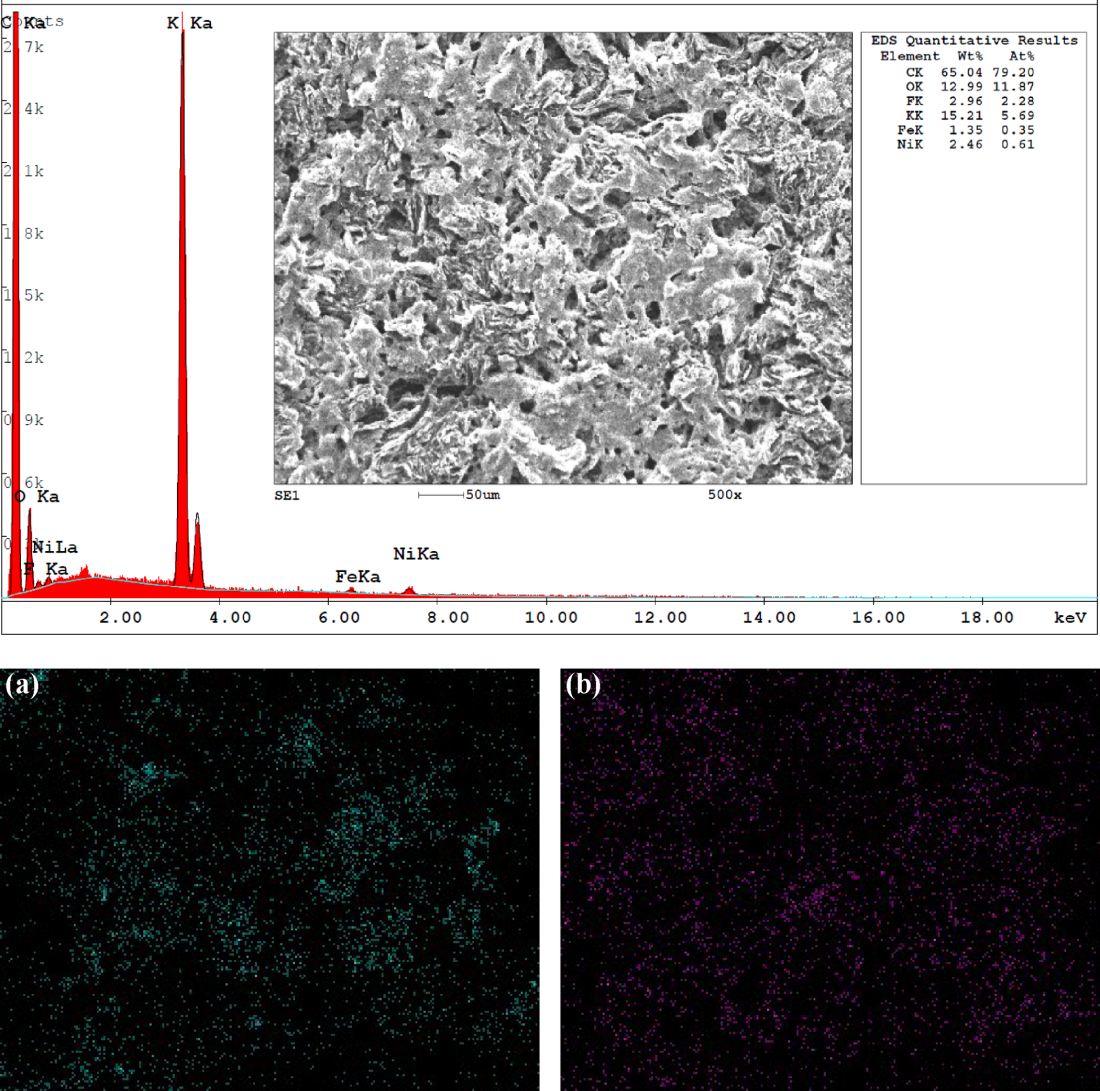
SEM image, EDX analysis, and elemental mapping for nickel (a) and iron (b) of a Ni/Fc-modified electrode surface after measuring its OER performance in 1 M KOH solution.

### 3.5 Tafel Slope, TOF, and Stability

The Tafel slope is another key parameter used to characterize the electrocatalysts for OER, providing information about its efficiency by indicating how much the overpotential needs to be increased to increase the reaction rate.^69^ In this work, it was performed by chronoamperometry measurements at the low overpotential region to avoid any contribution from the capacitance current and to reach a true steady state, unlike sometimes inaccurate slopes derived from linear scan voltammetry.^69^ Figure 10 depicts currents obtained after the application of different potentials and the Tafel plot obtained as a function of potential and overpotential versus log current density.

**Figure 10:**
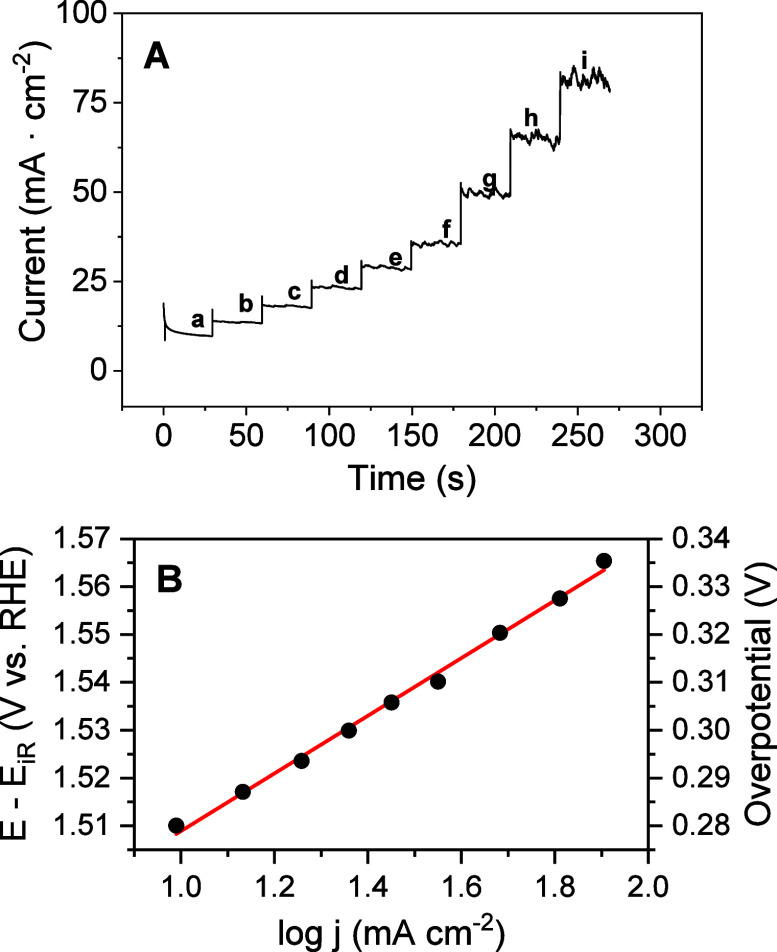
(A) Chronoamperometry measurements of a Ni/Fc-catalyst electrode in 1 M KOH. Potential applied: (a) 1.510; (b) 1.517; (c) 1.523; (d) 1.529; (e) 1.536; (f) 1.540; (g) 1.550; (h) 1.557; and (i) 1.565 V. (B) Tafel plot derived from current densities obtained in function of compensated potential applied and overpotential.

From the linear fitting, a Tafel slope value of 60 mV dec^–1^ was obtained, which lies in the typical range of values (30–120 mV dec^–1^) reported in the literature for electrocatalysts of Fe and Ni oxides/hydroxides.^70–72^ The reaction has been proposed to undergo oxidation from the 2^+^ state to the 3^+^ state of the metal by releasing only one electron per site:

$$M^{2+}-\mathrm{OH}+{\mathrm{OH}}^{-}\left(\mathrm{aq}\right)\to M^{3+}\left(O\right)-\mathrm{OH}+H^{+}\left(\mathrm{aq}\right)+e^{-}$$

$$M^{3+}\left(O\right)-\mathrm{OH}+{\mathrm{OH}}^{-}\left(\mathrm{aq}\right)\to M^{2+}-\mathrm{OH}+H^{+}\left(\mathrm{aq}\right)+O_{2}\left(g\right)+e^{-}$$

It leads to the dioxygen evolution by the simultaneous oxidation and formation of hydroxide and oxyhydroxide.^72^ This result suggests that the Ni/Fc electrocatalyst has relatively efficient kinetics, allowing the reaction to proceed with a lower overpotential.

TOF is another significant activity parameter in water splitting, which provides information on the kinetics of the OER for the catalytic material. It was calculated according to eq 1 and by quantifying the redox peaks of Ni^3+^/Ni^2+^ via integration of the cathodic peak measured by CV (Figure 1). A TOF value of 0.21 s^–1^ was obtained at an overpotential of 300 mV, which is higher than those reported for other Ni–Fe catalysts,^73^ suggesting a high intrinsic activity for our catalyst.

Stability is another important parameter to check whether the catalyst has the potential for use in practical water electrolysis applications, especially when it is exposed to highly corrosive and oxidative conditions. It was evaluated by carrying out chronopotentiometry experiments at current densities of 10 mA·cm^–2^ (considered benchmark for a 10% efficient solar-to-fuels conversion device performance) and 100 mA·cm^–2^ (large current density for industrial requirements), respectively. As can be seen in Figure 11A, the Ni/Fc catalyst provided a constant potential of ∼1.51 V and an overpotential of ∼ 0.278 V at 10 mA·cm^–2^ for more than 26 h, keeping the film stable without any loss of activity. In the case of 100 mA·cm^–2^ applied current density (Figure 11B), a potential of ∼1.65 V (i.e., an overpotential of ∼0.420 V) was measured for more than 46 h with only a slight increase of the potential at the beginning of the experiment, which further remains almost stable. The XRD diffraction pattern further obtained (Figure S8) confirmed the stability and preservation of the carbonaceous electrode.

**Figure 11:**
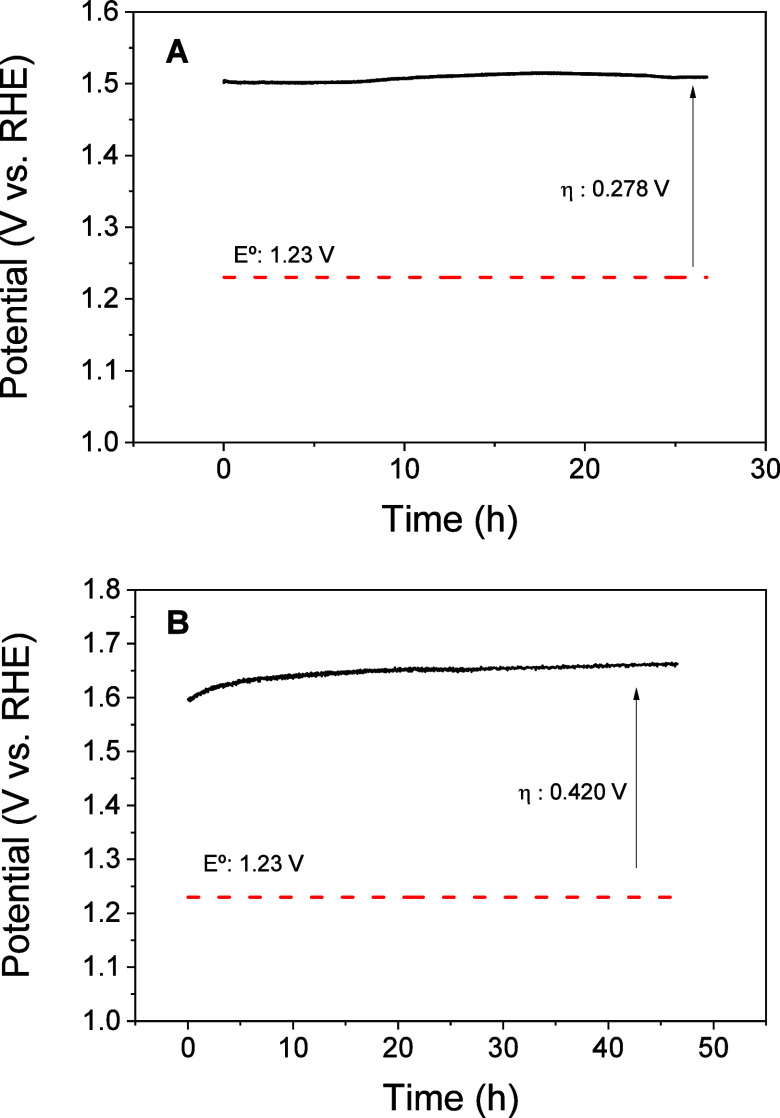
Chronopotentiometry stability test of Ni/Fc-modified electrodes for 26 h at 10 mA·cm^–1^ (A) and for 46 h at 100 mA·cm^–1^ (B) in 1 M KOH solution.

A comparison of different catalysts reported in the literature based on their overpotentials and Tafel slopes is included in Table S1, where it is seen that our Ni/Fc catalyst provided lower or similar overpotentials in comparison to other efficient bimetallic NiFe catalysts reported. Additionally, Wang et al.^74^ reported a methodology to compare OER catalysts based on their overpotentials and operational stabilities in which our catalyst lies in the category of ideal.

## 4 Conclusions

The combination of Ni/Fc leads to a synergistic effect similar to that we recently reported for Co/Fc, but with much better results. It constitutes a straightforward way for obtaining OER catalysts showing high current densities, lower overpotential, high stability, and low Tafel slope in alkaline conditions. The method is simple, allowing the preparation of anodes easily without the need for previous and tedious synthetic procedures, as only commercial chemicals with low amounts of metals are employed and thus can be scaled up to real devices.

## Supplementary Material



## Abbreviations

OER: oxygen evolution reaction
Fc: ferrocene
LSV: linear sweep voltammetry
SEM: scanning electron microscopy
CV: cyclic voltammetry
EIS: electrochemical impedance spectroscopy
XPS: X-ray photoelectron spectroscopy
XRD: X-ray diffraction analysis
