# Supplementary material for: Nickel and Ferrocene as Catalyst Candidates to Promote an Effective Oxygen Evolution Reaction
Source: ACS Omega. 2025 May 7;10(19):19552–63. doi: 10.1021/acsomega.5c00165 (PMC12096218; doi:10.1021/acsomega.5c00165)
Supplement: Supplementary file 1 [file ao5c00165_si_001.pdf]

Supporting Information

Nickel and ferrocene as catalyst candidates to promote an effective oxygen evolution reaction

Jose M. Abad, María Victoria Martínez-Huerta, Jesús Cebollada, Raquel Sainz, Marcos Pita and Antonio L. De Lacey*

Instituto de Catálisis y Petroleoquímica, CSIC. C/Marie Curie 2, 28049 Madrid, Spain.

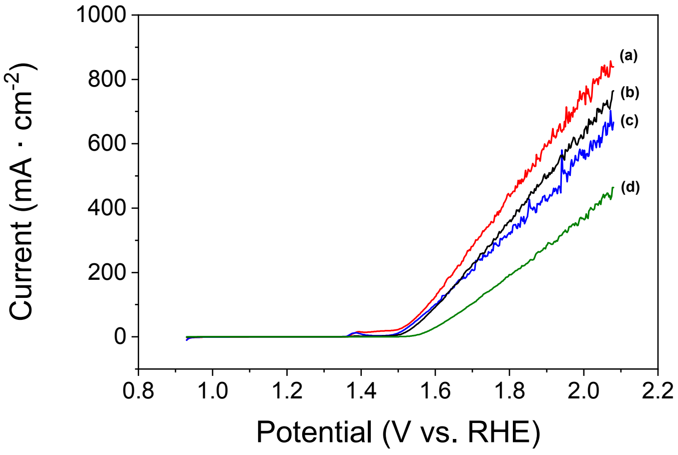

Figure S1. Linear sweep voltammograms in 1 M KOH solution of Ni/Fc-modified electrodes with different $NiCl_2 \cdot 6H_2O$ loadings: (a) 36 µg, 0.5 mg·cm^{-2}; (b) 18 µg, 0.25 mg·cm^{-2}; (c) 72 µg, 1 mg·cm^{-2}; (d) 9 µg, 0.125 mg·cm^{-2}. All catalysts contain Ferrocene (18 µg, 0.25 mg·cm^{-2}). Scan rate, 5 mV·s^{-1}.

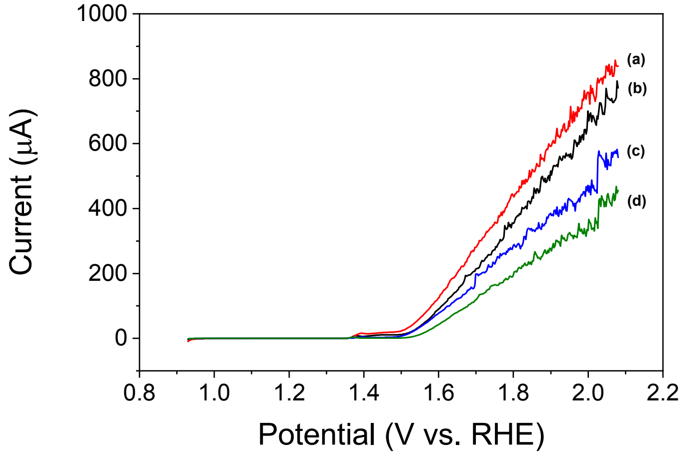

Figure S2. Linear sweep voltammograms in 1 M KOH solution of Ni/Fc-modified electrodes with different Ferrocene loadings: (a) 18 µg, 0.25 mg·cm^{-2}; (b) 36 µg, 0.5 mg·cm^{-2}; (c) 9 µg, 0.125 mg·cm^{-2}; (d) 5.4 µg, 0.075 mg·cm^{-2}. All catalysts contain $NiCl_2 \cdot 6H_2O$ (36 µg, 0.5 mg·cm^{-2}). Scan rate, 5 mV·s^{-1}.

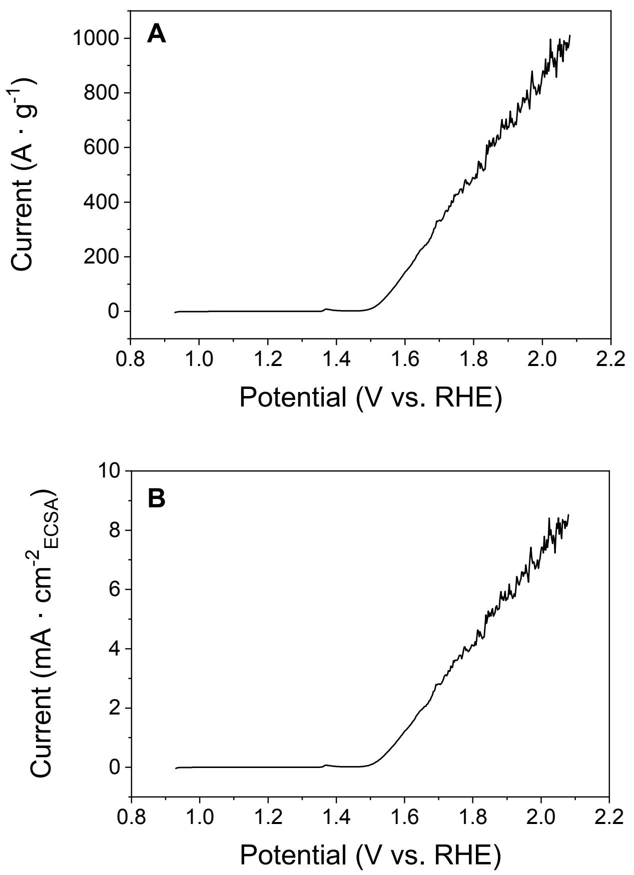

Figure S3. OER LSVs of a Ni/Fc -catalyst electrode in 1 M KOH at a scan rate of 5 mV·s^{-1} with current normalized by: (A) mass loading of $NiCl_2 \cdot 6H_2O$ and Fc; (B) ECSA.

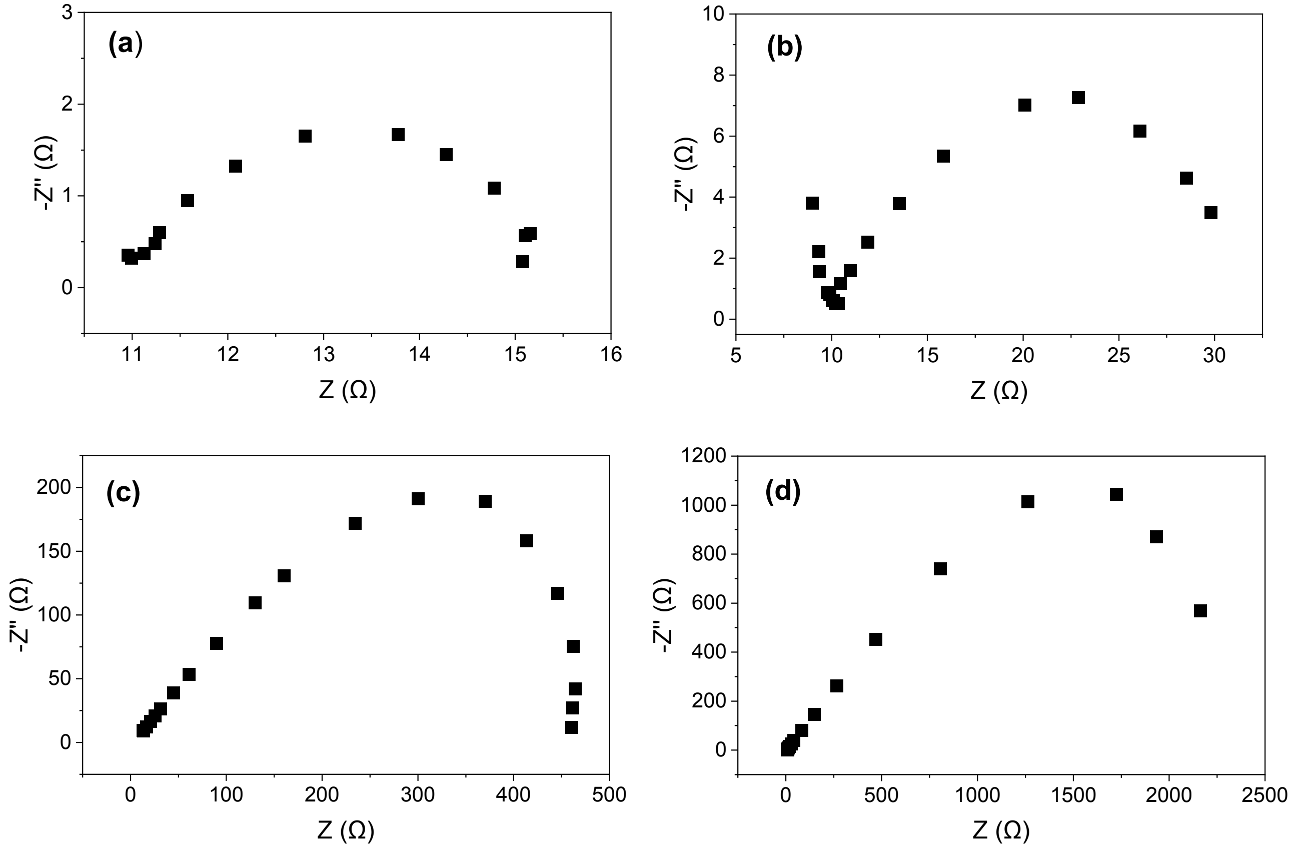

Figure S4. EIS measurements of a Ni/Fc-catalyst (a); Ni-catalyst (b); Fc-catalyst (c); Vulcan (d) electrodes in 1 M KOH.

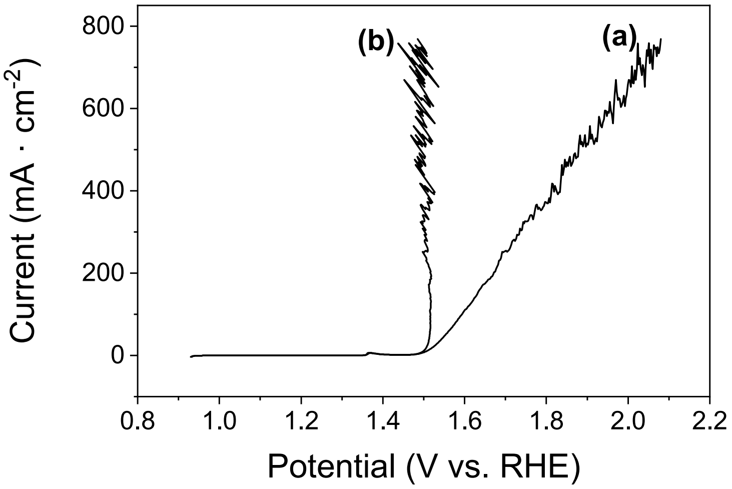

Figure S5. LSVs of a Ni/Fc -catalyst electrode in 1 M KOH at a scan rate of 5 $mV \cdot s^{-1}$ without (a) and with 100% iR drop compensation (b).

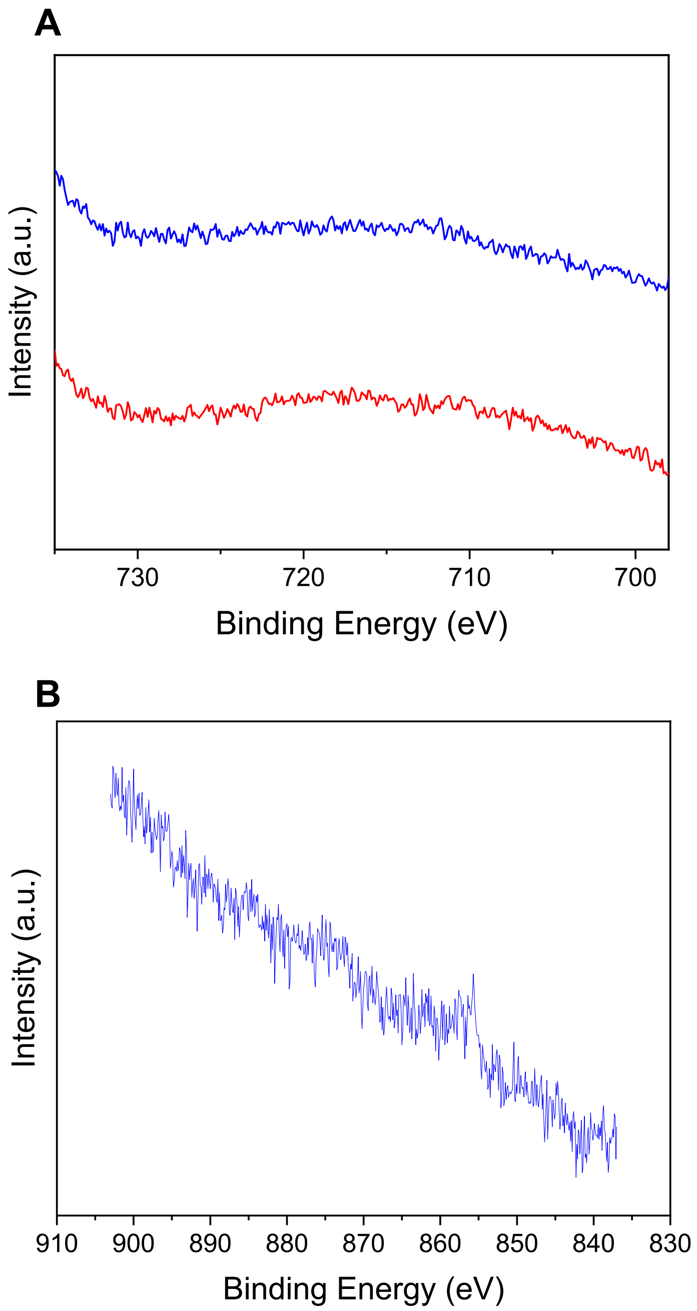

Figure S6. High resolution XPS spectra for: (A) Ni/Fc-modified electrodes; Fe 2p energy region: Electrodes as-prepared before any electrochemical process (curve red); after studying their OER performance (curve blue). (B) Ni-modified electrode; Ni 2p energy region after studying their OER performance.

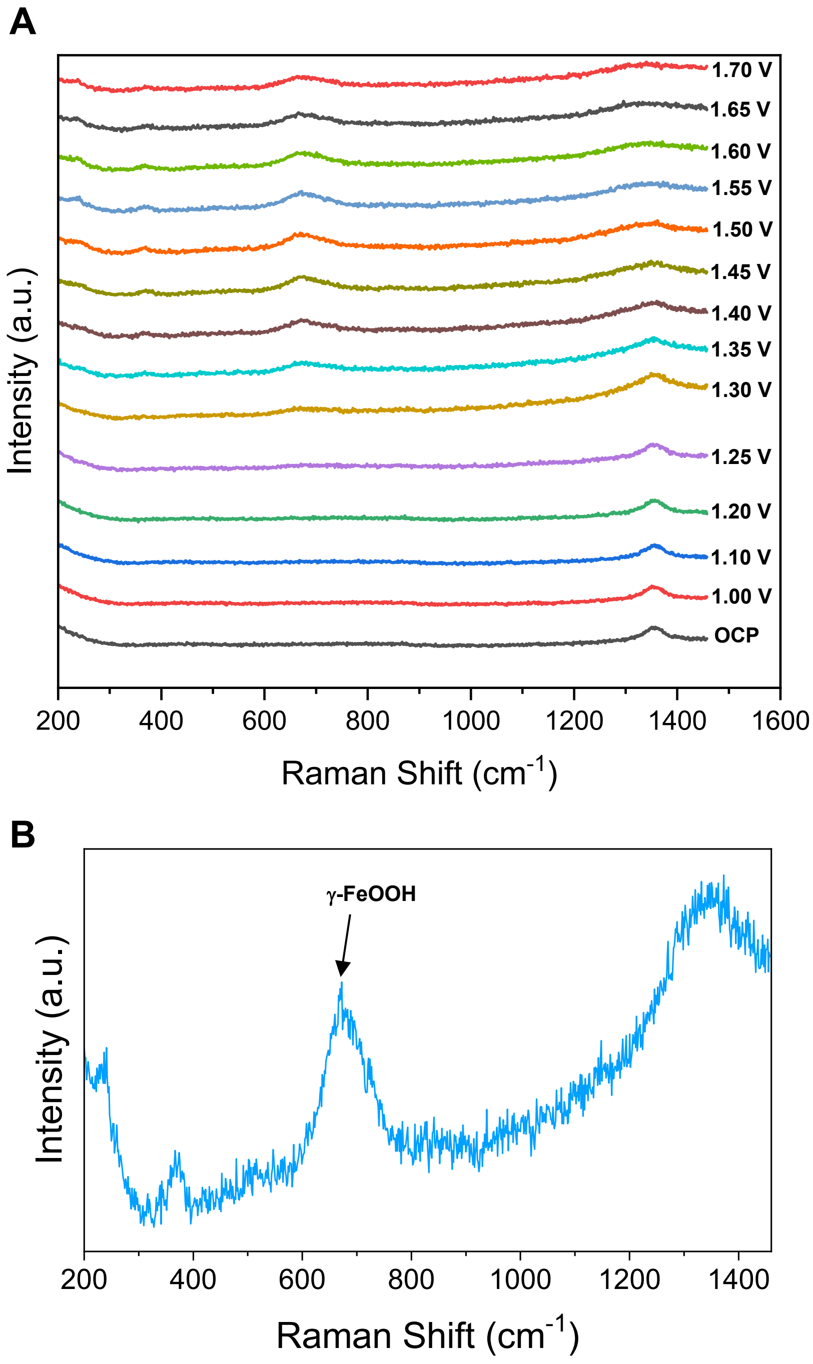

Figure S7. In situ Raman spectra collected for Fc-modified electrode as function of potential applied vs. RHE in 0.1 M KOH (A) and (B) zoomed spectrum obtained at 1.55 V.

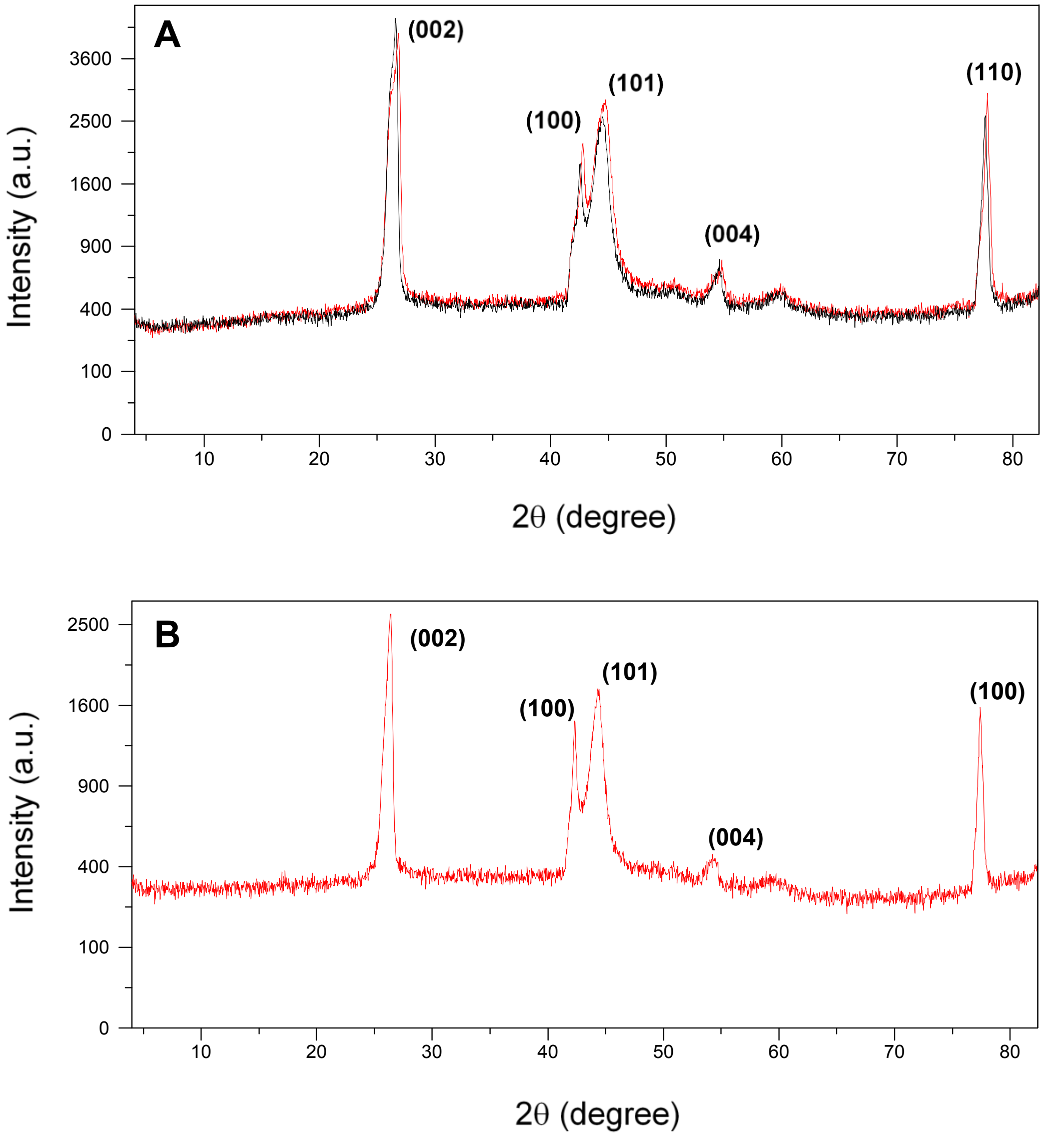

Figure S8. XRD analysis of: (A) Ni/Fc-catalyst electrode as-prepared (red line) and after studying their OER performance (black line) for more than 45 hours at 100 mA·cm^{-2} in 1 M KOH solution; (B) Vulcan-

modified electrode. Bands are indicative of the graphite crystal structure: (002) corresponds to the interlayer spacing between the graphene layers; (100) and (101) are related to the in-plane structure of the graphite lattice; (004) second-order reflection of the (002) plane; (100) corresponds to the in-plane lattice spacing.

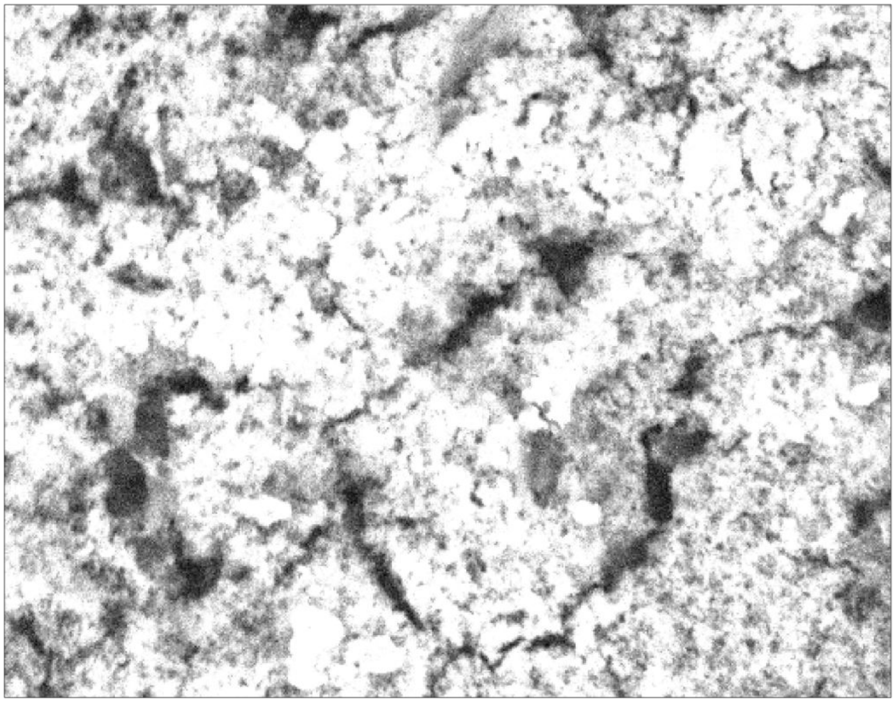

SE1 10um 2400x

EDS Quantitative Results		
Element	Wt%	At%
CK	52.84	70.08
OK	19.60	19.51
KK	21.35	8.70
FeK	1.72	0.49
NiK	4.49	1.22

C:\EDAX32\GENESIS\GENMAPS.SPC

kV:20.0	Tilt:0.00	Tkoff:31.84	Reso:137.54	Amp.T:25.6
FS : 1462	LSec : 29.0	Prst:None	25-Oct-2024	12:17:15

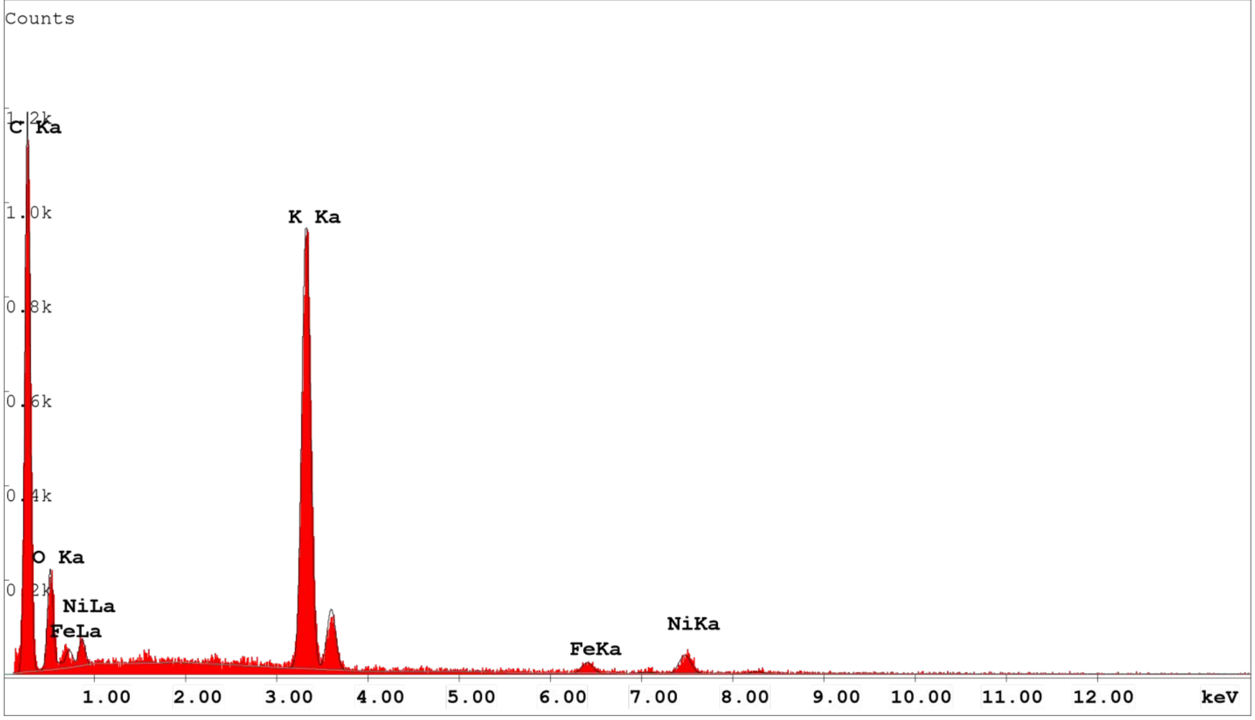

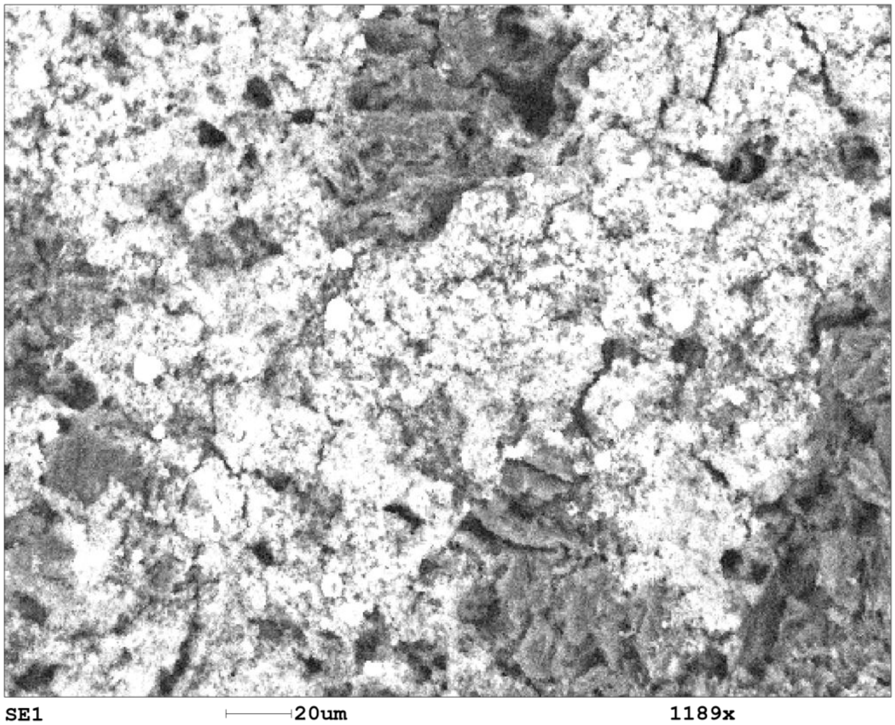

EDS Quantitative Results

Element	Wt%	At%
CK	50.94	68.99
OK	19.52	19.84
KK	21.19	8.82
FeK	2.47	0.72
NiK	5.88	1.63

C:\EDAX32\GENESIS\GENMAPS.SPC

kV:20.0	Tilt:0.00	Tkoff:31.84	Reso:137.54	Amp.T:25.6
FS : 1262	LSec : 28.6	Prst:None	25-Oct-2024	12:11:00

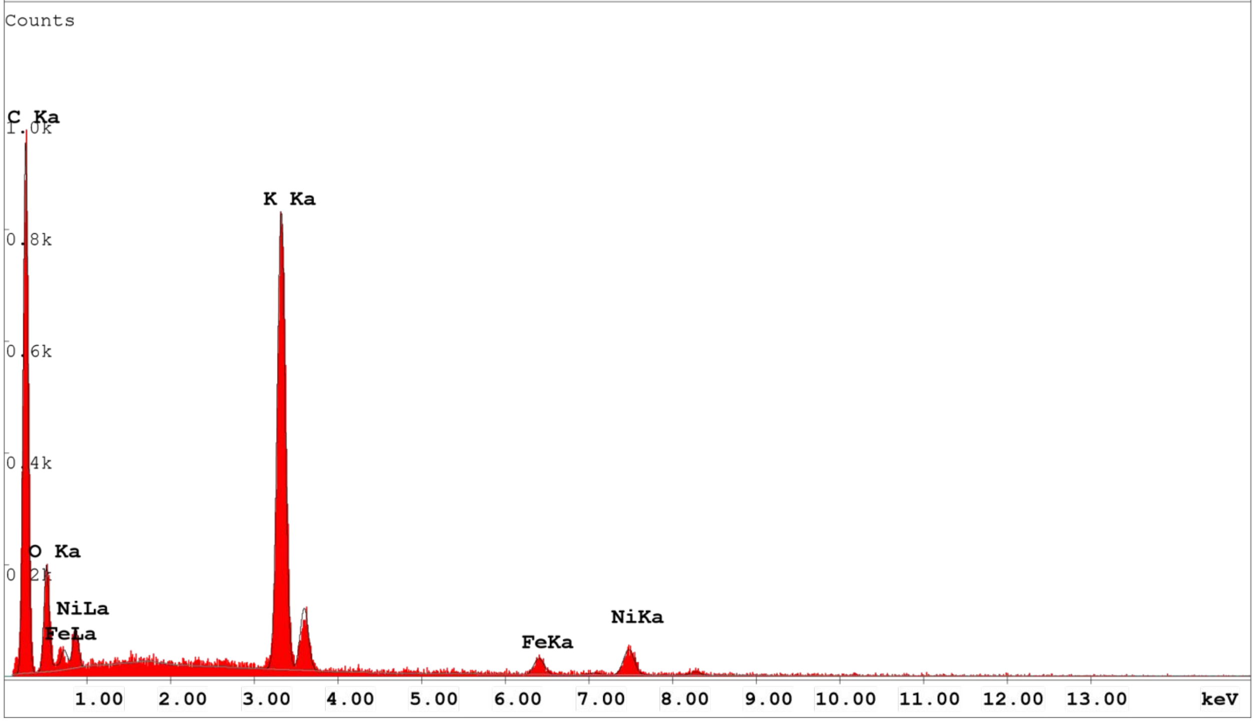

Figure S9. SEM images and EDX analysis of Ni/Fc-catalyst electrode surfaces after OER performance in 1 M KOH solution.

Table S1

OER activity, types of electrodes/electrolytes, Tafel slope and overpotential at 10 mA cm^{-2} of typical materials reported in the literature.

**Catalyst**	**Electrolyte**	**Electrode**	**η (mV) @ 10 mA cm^{-2}**	**Tafel slope ($mV \cdot dec^{-1}$)**	**Reference**
FeNi Alloy nanoparticles	1 M KOH	Carbon fibers	317	49	Wei et al., *Journal of Colloid and Interface Science,* 2020, *578,* 805.
IrO_2	1 M KOH	Glassy carbon	338	47	Hu et al., *Nat. Commun.,* 2014, **5,** 4477.
$Ni_{0.9}Fe_{0.1}Ox$	1 M KOH	ITO	336	30	Boettcher et al., *J. Am. Chem. Soc.* 2012, **134**, 17253–17261.
NiFe-LDH nanosheets	1 M KOH	Glassy carbon	300	40	Hu et al., *Nat. Commun.,* 2014, **5,** 4477
$Ni_xFe_{1-x}S$	1 M KOH	Ni foam	122	120	Han et al. *Applied Catalysis B: Environmental,* 2022, **304**, 120937.
**Ni/Fc-catalyst**	1 M KOH	Graphite	270	60	This work
Nickel foam anodized + Fe (III)	1 M KOH	Nickel foam	242	35	Hu et al. *Inorg. Chem.,* 2024, **63**, 15493.
FeNi (1:1 Ni/Fe) alloy	1 M KOH	Nickel foam	270	35	Akbari et al., *Langmuir,* 2023, **39**, 11807.
Fe-doped NiOOH	1 M KOH	Nickel foam	240	62	Li et al., *Materials,* 2024, **17**, 4670.
NiFe-CNG	1 M KOH	Graphitic carbon nitride	270	69	Wan et al. *Nat. Commun.,* 2021, **12**, 5589.

Ni-N4/GHSs/Fe-N4	0.1M KOH	Graphene hollow nanospheres	390	81	Ma et al. *Adv. Mater.* 2020, **32**, 2003134.
FeNiF/NCF	1 M KOH	Nitrogen-doped porous nanofibers	260	67	*Zha et al., Journal of Energy Chemistry,* 2020, **47,** 166.
Fe NCs|NiO NS<br>Fe nanoclusters on nickel oxide	1 M KOH	Nickel electrode	380	111	Maduraiveeran et al., *Langmuir,* 2024, **40**, 22549.
